# Oral health and oral health-related quality of life in people with Marfan syndrome: a cross-sectional study

**DOI:** 10.1186/s12903-025-06318-2

**Published:** 2025-06-05

**Authors:** Nick Jan Tetsch, Jeanette Köppe, Linda Daume, Johannes Kleinheinz, Marcel Hanisch, Ole Oelerich

**Affiliations:** 1 Department of Cranio- and Maxillofacial Surgery, University Hospital Münster, Albert-Schweitzer-Campus 1, Building W30, D-48151 Münster, Germany; 2 Institute of Biostatistics and Clinical Research, University of Münster, Münster, Germany

**Keywords:** Marfan syndrome, Oral health-related quality of life, Rare diseases, OHIP-G5, PhOX

## Abstract

**Background:**

Marfan syndrome is a rare connective tissue disorder. This study was initiated to investigate the impact of Marfan syndrome on oral health.

**Methods:**

The person-reported perceived OHRQoL was determined using the German short version of the Oral Health Impact Profile (OHIP-G5). In addition, all study participants took part in a clinical examination in which the objective oral health was measured using the Physical Oral Health Index (PhOX).

**Results:**

A total of 29 patients took part in the study. Of these, 20 were female and nine male and the median age was 54 years (interquartile range (IQR): 15). The median OHIP-G5 score was three (IQR: 5) (range: 0–15). The median PhOX score was 79 (IQR: 14.5). A statistically significant correlation between the OHIP-G5 and the PhOX could not be determined (*r*=-0.34, *p* = 0.074).

**Conclusions:**

The study showed that OHRQoL in particular was reduced. The measured oral health was slightly reduced compared to the general population. Nevertheless, Marfan patients should pay great attention to oral hygiene which should be checked by dental professionals at regular intervals.

**Clinical trial number:**

Not applicable.

## Background

Marfan syndrome is an autosomal dominant inherited systemic connective tissue disorder and can manifest itself in different ways [1]. Marfan syndrome is a rare disease with an incidence of one in 5.000 individuals. The term “rare disease” is used when the prevalence of the condition is less than one in 2.000 individuals [2]. Both sexes are equally affected [3]. Due to the mutation in the gene for the protein fibrillin-1 (FBN1) on chromosome 15q21, many parts of the body are affected [4]. Pathologies usually occur in the eye region, the skeleton and the cardiovascular system [5, 6] including pain as a significant problem associated with profound disability and psychological burden [7]. As there is no specific laboratory test except molecular genetic testing to diagnose Marfan syndrome [8], the diagnosis is made on the basis of clinical criteria defined in the 2010 Ghent Nosology [9, 10]. Oral health is precisely defined by the WHO [11]. The current study situation on the effects of Marfan syndrome on oral health is not clear. A systematic review from 2019 found no statistically significant correlation between Marfan syndrome and oral health status [12], while another study from 2013 found no statistically significant differences in the periodontal situation between people with Marfan syndrome and the control group [13]. In contrast, several other studies were able to identify a possible change in the mucosa, periodontium, dentition, jawbone and dysfunction with the temporomandibular joint [13, 14]. Anatomically, a retrognathia of the lower jaw and a high arched, narrow palate are described [15, 16]. The anatomy of the palate by its narrow and elevated structure in patients with Marfan syndrome, has been identified as a potential contributing factor to the development of posterior crossbite [17]. The long narrow face and the narrow maxilla can lead to considerable crowding of the teeth and to malocclusion [17]. The teeth may also show abnormalities. For example, incomplete development and crown dysplasia have been described [18].

In an online questionnaire of the research group preceding this study, it also became clear that the OHRQoL is reduced in Marfan patients [19]. In light of the aforementioned background, the objective of the present study was to undertake a clinical examination of patients suffering from Marfan syndrome. To this end, the person-reported perception of the participants was recorded using questionnaires. The development of the used OHIP-questionnaire is rooted in the theoretical underpinnings established by Locker in 1988. His seminal work involved the formulation of a model grounded in the World Health Organization´s conceptualization of the impact of disease. This model yielded five key consequences for oral health encompassing impairment, functional limitation, pain/discomfort, disability, and handicapping [20]. Standardized tests were employed to investigate the extent to which there is a connection between oral health-related quality of life (OHRQoL) and objective oral health. The potential for disparity between objective and person-reported oral assessment arises from divergent evaluation criteria employed by practitioners and patients. In the case of patients, the outcomes of standardized diagnostic questionnaires often take a secondary role, while functional, psychological, and social transformations hold greater significance [21].

In light of the inconclusive findings of the aforementioned studies, the objective is to conduct a more exhaustive investigation into the oral health of Marfan patients. A primary focus of this investigation will be to ascertain the existence of a significant correlation between self-reported and objective assessments of oral health. This study is expected to offer further insight into the impact of this rare disease on oral health.

## Materials and methods

The Ethics Committee of Westfalen-Lippe in Münster, Germany approved the application for the study in January 2024. The data were collected from March 2024 to June 2024, from Marfan patients completing a questionnaire prior to clinical investigation.

### Recruitment

Participants in the study were informed about the study project in February via the Marfan Hilfe e.V. website (www.marfan.de) and the Marfan Hilfe e.V. Facebook page. The study was also presented at the Marfan Day 2024 in Fulda (May 10–12, 2024) in order to reach and examine as many affected people as possible.

### Inclusion criteria

The inclusion criteria for participation in the study were as follows: Participants had to be at least 18 years old. They had to have provided written informed consent to take part in the study. A confirmed diagnosis of Marfan syndrome according to the 2010 Ghent nosology was required. In addition, participants had to be able to speak and write German.

### Exclusion criteria

Pregnant women, minors, people with mental disabilities, and people with legal representatives were excluded from the study. A multilingual implementation would necessitate additional organizational and personnel costs, which could not be guaranteed within the scope of the data collection. This also minimizes bias due to language barriers and ensures greater validity.

### Data collection

The data collection, which was anonymized, was divided into a questionnaire part, where the participants had to fill out the questionnaire, and the participation in a clinical examination. The questionnaire collected general patient-related data (age, sex, age at diagnosis of Marfan syndrome, time between first symptoms and diagnosis, general symptoms, medication) and oral health-related quality of life using the German short form of the Oral Health Impact Profile (OHIP-G5 [22]). During the clinical examination, measures of oral health were determined using the Physical Oral Health Index (PhOX [23]). The anonymity of the participants was ensured by coding the questionnaires in such a way that they could be assigned to each other by using numbers. Nevertheless, it is not possible to trace them back to the participants. The use of X-ray images was ruled out due to considerations regarding feasibility and the need for X-ray protection. In order to ensure consistent data collection, X-ray images would have had to be taken for all patients, but in most cases there is no clinical indication for taking such images, which is why this option was rejected.

### Oral health-related quality of life

To determine OHRQoL, the OHIP-G5 questionnaire was used. The questionnaire consists of five questions about the dimensions of oral health, oral function, orofacial pain, orofacial aesthetics and psychosocial influence. The responses were given on a Likert-scale, ranging from [4] very often [3], often [2], sometimes [1], hardly ever to (0) never, resulting in a range between 0 and 20 points for the OHIP-G5 summary score. The fewer points calculated in the evaluation, the better the person-reported oral health of the participant, additionally it is important to note that the study participants completed the questionnaire independently before the clinical examination. Besides the summary score, individual scores for each dimension of OHRQoL were calculated as currently recommended [24]. The “oral function” dimension is the sole one that comprises two questions. For comparability, the two values for the dimension “oral function” were added and divided by two, to result in a dimension score ranging from zero-four for all dimensions [25].

### Physical oral health index (PhOX)

Oral health was assessed using the PhOX questionnaire which is divided into four parts. The PhOX covers 14 areas relevant to oral health which can be assessed objectively and systematically using the questionnaire. In categories one to four the teeth and surrounding tissue are examined. Categories 5 to 7 examine the intraoral soft tissue, categories 8 to 10 the soft tissue and the jaw and categories 11 to 14 determine function and perception.

During the intraoral examination, the examiner determines the dental findings, differentiating between healthy, filled/crowned teeth, decayed/defective teeth, replaced teeth, missing teeth and gap closure. The periodontium is examined by measuring the probing depth on the Ramfjord teeth [26] (16, 21, 24, 36, 41, 44) and examining bleeding on probing. For accurate evaluation, pocket depth was measured at six points per tooth. The measurement was conducted using a World Health Organization (WHO) probe, which has markings at 3.5 mm, 5.5 mm, 8.5 mm and 11.5 mm and thus enables precise measurement of pocket depths. In the endodontic region, the teeth are checked for pain on percussion and by means of the tapping sound. The number of support zones, orthodontic aspects (overjet, overbite, crossbite) and the mouth opening are assessed. The quality and quantity of saliva are evaluated through a visual assessment of the moistening of the oral mucosa, lips, and tongue. The classification system employed to assess saliva includes the following categories: moist, partially dry and completely dry. The consistency of saliva is also evaluated, with the classification system including the following: liquid, slightly foamy, and no saliva. In addition, it is inspected whether there are any gaps or defects and whether there is any swelling, discoloration or loss of integrity. The mouth opening was determined using a Beerendonk caliper and is determined by the overbite and the distance between the incisal edges. Each category is scored between four and zero, with four representing the best oral health in this category. The determination is made on the basis of predefined criteria [23]. Each of the 14 sub-items was given a single, double or triple weighting, depending on its importance, so that a score of 100 represents the best possible objective oral health according to the PhOX scoring recommendation [23] (Table 1). The examination was carried out by two different examiners. These examiners had previously undergone training and calibration in conjunction with an experienced user of the PhOX at University Hospital Münster.

**Table 1:** Physical oral health index (PhOX) weights and ranges for each item

Item	Weight	Range
Number of teeth	3	0–12
Tooth structure	3	0–12
Periodontium	3	0–12
Endodontia	2	0–8
Surface	1	0–4
Color	2	0–8
Moisturization	1	0–4
Pain of palpation	1	0–4
Continuity	1	0–4
Proportion	1	0–4
Mouth opening	1	0–4
Supporting Area	3	0–12
Pain	2	0–8
Paresthesia	1	0–4

### Statistical methods

The study was conducted descriptively, and no a priori power analysis was performed. General demographic data (e.g., age, sex, age at diagnosis, and time between first symptoms and diagnosis) as well as data on the OHIP-G5 and the PhOX were collected and analyzed. Descriptive statistics were used to summarize the data, and results were visualized using tables and graphs.

To explore sex-specific differences in OHIP-G5 and PhOX scores, a Mann-Whitney U test was applied with a significance level of 5%. For the assessment of correlations between PhOX and OHIP-G5, a Spearman correlation coefficient was calculated. Statistical analyses were conducted using SPSS version 29.0.2.0 (IBM Corp., Armonk, NY, USA) and RStudio version 2022.07.1 + 554 (RStudio PBC, Boston, MA, USA) for graphs.

## Results

### Age and diagnostic period

In total, 20 (69%) female participants and nine (31%) male participants took part in the study, bringing the number of participants to 29. No one had to be excluded from participating in the study. The median age of the study participants at the time of data collection was 54 years (interquartile range (IQR: 15)), with the youngest participant being 25 years old and the oldest person 71 years old. The median age at diagnosis was 29 years (IQR: 31). It took a median of four years (IQR: 20) between the first symptoms and the diagnosis, with the maximum duration being 38 years. The median age of female participants at diagnosis was nine years higher than that of male participants, the median time between the first symptoms and diagnosis was three years longer for female participants than for male participants.

Detailed information on the demographic data can be found in Table 2.

**Table 2:** Overview of demographic patient data

	*n* (%)	Mean (SD)	Median (IQR)	Range in years
Age		51.7 (± 11.1)	54.0 (15.0)	46
*Male*		53.4 (± 9.2)	56.0 (14.0)	28
*Female*		51.0 (± 12.0)	52.5 (15.0)	46
Sex				
Male	9 (31%)			
Female	20 (69%)			
Age at diagnosis		23.9 (± 16.9)	29.0 (31.0)	56
Male		20.7 (± 15.4)	21.0 (31.0)	
Female		25.3 (± 17.7)	30.0 (15.0)	
Time between first symptoms and diagnosis		10.2 (± 12.1)	4.0 (20.0)	38
Male		5.6 (± 8.5)	2.0 (7.0)	24
Female		12.0 (± 13.1)	5.0 (23.0)	38

### OHRQoL

**Fig. 1:**
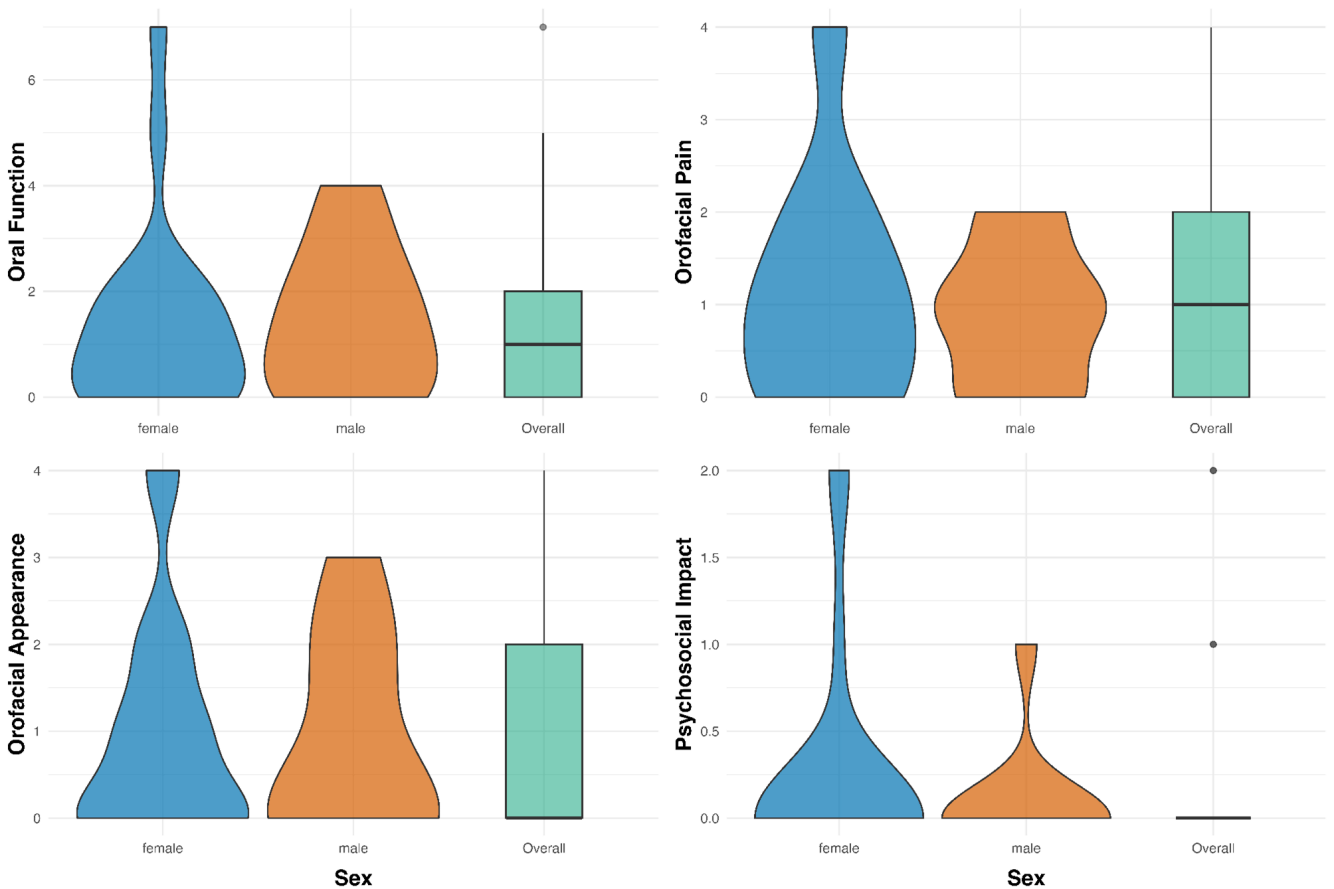
Violin Plot for each dimension of OHRQoL

In the evaluation of the OHIP-G5 questionnaire, a median of three points (IQR: 5) was obtained for the overall evaluation. Male participants had a lower total score than female participants. However, the difference between the results of male and female participants are not statistically significant (*p* = 0.982). The results of the sub-items (oral function, orofacial pain, orofacial aesthetics, psychosocial influence) are presented in Table 3; Fig. 1. Figure 1 shows a violin plot, which is a useful tool for data visualization, offering a comprehensive representation of the distribution of data in small sample sizes. Since this type of representation is based on density functions, the distribution within smaller groups can be better represented, allowing for a quicker assessment of whether symmetrical data is present or whether there are many outliers in the sample.

**Table 3:** Results (mean + SD) of the 4 sub-items of the OHIP-G5 and total score

	Oral function	Orofacial pain	Orofacial esthetics	Psychosocial influence	OHIP-G5 Total score
Total (*n* = 29)	1.4 (± 1.7)	1.1 (± 1.1)	0.9 (± 1.2)	0.2 (± 0.6)	3.6 (± 3.6)
Male	1.4 (± 1.4)	0.9 (± 0.8)	0.9 (± 1.2)	0.1 (± 0.3)	3.3 (± 2.8)
Female	1.4 (± 1.8)	1.2 (± 1.2)	1.0 (± 1.3)	0.3 (± 0.6)	3.7 (± 4.0)
*p*-values	**0.660**	**0.799**	**0.945**	**0.835**	**0.982**

### Oral health

In the study collective, a little more than one third of the participants had complete dentition. The median number of the teeth was between 21 and 27 (IQR: 1). Only one of the people had no need for treatment on any tooth. A larger portion of participants had more healthy than filled teeth and no need for treatment. Slightly fewer participants had more filled than healthy teeth and no need for treatment. Nearly half of the participants showed more than three teeth with a probing depth of 3.5–5.5 mm or one-three teeth with a probing depth of > 5.5 mm. Endodontically, the majority of the subjects did not have a tooth with tapping pain. Only one participant had an extensive change in the oral mucosa, the rest showed no changes in the endodontic assessment. The majority showed no percussion sensitivity. Changes in the oral mucosa were extremely rare. Only one person showed a marked alteration, while the rest were clinically unremarkable. The color and texture of the oral mucosa, lips, and tongue were mostly rosy and free of swelling. Mild or severe alterations appeared only in isolated cases, and always without swelling. Salivary flow was unremarkable in most participants. They showed liquid saliva and fully moist mucous membranes. Some of the participants had partial oral dryness, accompanied by either liquid or slightly foamy saliva. Slightly more than the half of the study population reported no pressure tenderness (salivary glands, muscles, temporomandibular joint), two had mild unknown pain on pressure, six had moderate unknown pain or mild known pain, and a further four reported severe unknown pain or moderate known pain. All but one subject had no cleft or deficiency (jaw, palate, tongue, lip), the one subject had a cleft or deficiency of soft tissue. The size ratio (jaw, tooth position) showed a variable appearance, 20 participants had a score of 4 (0 deviations), in the mouth opening only eight had a mouth opening ≥ 40 mm straight, the percentage majority had a mouth opening between 30 and 39 mm. Only one person examined did not have four support zones, but only three. Pain was of varying severity. Between none and very often pain was rarely (24%), occasionally (28%) and often (3%) represented.

There were also varying degrees of discomfort (very often: 14%, often: 7, occasionally: 3%, rarely: 14%, none: 62%).

The median total PhOX was 79 (IQR 14.5). Male participants had a median score of 79 (IQR: 12) and female participants of 78 (IQR: 15).

Detailed information can be found in Table 4.

**Table 4:** Mean values for each phox (Physical oral health Index) domain in total and for individual sexes

	Total (*n* = 29)	Male (*n* = 9)	Female (*n* = 20)
Teeth quantity	3.3 (± 0.6)	3.6 (± 0.5)	3.3 (± 0.6)
Condition of teeth	2.3 (± 1.0)	2.3 (± 0.7)	2.3 (± 1.1)
Condition of periodontium	1.6 (± 0.9)	1.2 (± 0.4)	1.8 (± 1.0)
Condition of endodontium	3.7 (± 0.8)	3.4 (± 1.3)	3.8 (± 0.4)
Surface of oral mucosa	3.9 (± 0.6)	3.7 (± 1.0)	4.0 (± 0.0)
Color of oral mucosa	3.7 (± 0.8)	3.9 (± 0.3)	3.7 (± 0.9)
Moistening of oral mucosa	3.5 (± 0.9)	3.2 (± 1.3)	3.7 (± 0.7)
Pain on palpation of jaws and muscles	3.1 (± 1.2)	3.8 (± 0.7)	2.8 (± 1.2)
Continuity of jaws, palate and tongue	3.9 (± 0.4)	4.0 (± 0.0)	3.9 (± 0.4)
Size ratio of jaw	3.6 (± 0.6)	3.4 (± 0.5)	3.7 (± 0.7)
Mouth opening capacity	3.2 (± 0.6)	3.2 (± 0.4)	3.2 (± 0.6)
Number of supporting zones	4.0 (± 0.2)	4.0 (± 0.0)	4.0 (± 0.2)
Pain frequency	2.8 (± 1.2)	3.2 (± 0.7)	2.7 (± 1.3)
Paresthesia frequency	3.0 (± 1.5)	3.7 (± 0.5)	2.8 (± 1.7)

### Correlation between ohrqol and oral health

In addition to the descriptive evaluation of the OHIP-G5 questionnaire and the PhOX questionnaire, no statistically noticeable correlation between both could be observed (*r*=-0.34, *p* = 0.074).

**Fig. 2:**
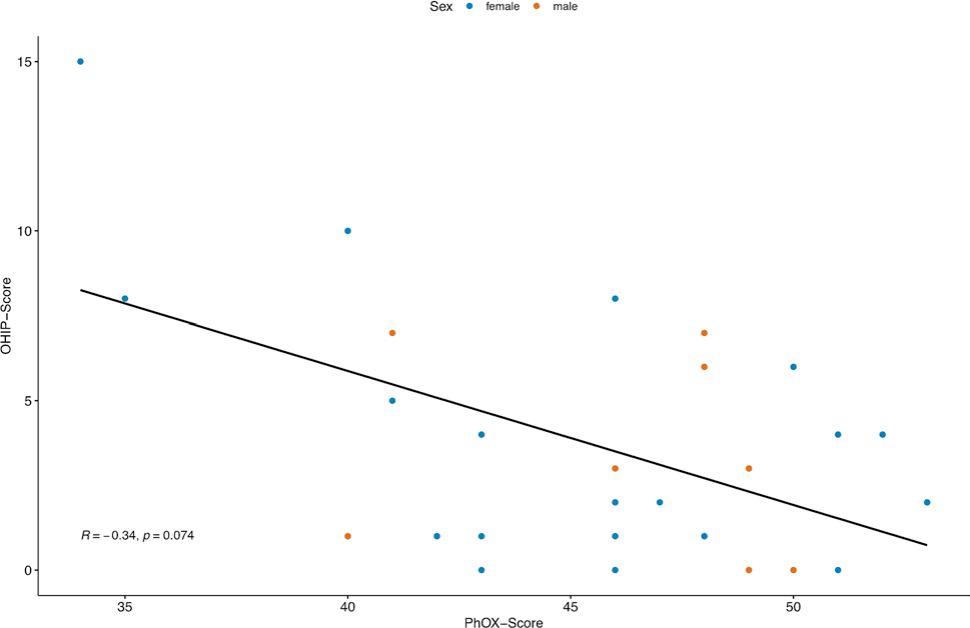
Scatterplot for correlation between PhOX (Physical Oral Health Index) and OHIP (Oral Health Impact Profile) scores with regression line; Sex is coded by color

Detailed information can be found in Fig. 2.

## Discussion

The aim of this study was to gain information on person-reported OHRQoL and objectively measured oral health. To the best of the author´s knowledge, this is the first study to explore this particular topic in the context of Marfan syndrome. In addition, the results are compared with other studies dealing with Marfan syndrome and oral health. This study is of interest to individuals suffering from Marfan syndrome and their relatives, as well as to dental professionals who often have limited knowledge about rare diseases [27].

The results for age, time of diagnosis and time between the first symptoms and diagnosis are comparable with those from the study by Jenabzadeh et al. [19] in which the median age of patients was 47 years (IQR: 16), the median age at diagnosis was 26 years (IQR: 27) and the time between the first symptoms and diagnosis was six years (IQR: 23). The extended period between the time between the first symptoms and diagnosis can be a source of distress for patients, largely due to the inherent uncertainty associated with this time frame [28]. Furthermore, it is plausible that individuals affected by Marfan syndrome may not receive specialized dental diagnosis or treatment for an extended period, which could potentially dimmish their OHRQoL.

The OHIP questionnaire is a scientifically accepted tool for measuring Oral Health-related Quality of Life. It measures the extent to which dental and oral health problems affect daily life, for example through pain, functional limitations, or social impairment. The advantages of the OHIP are its widespread use in practice and its international applicability [29].

Previous studies have already shown that OHRQoL can be markedly reduced in people with rare diseases [28, 30]. The OHIP-G5 was developed to provide a shorter and more pleasant variant for the patient than the previous OHIP variants especially for studies and screenings when time and effort are limited, with 90% of the information from the OHIP-G49 contained and summarized in the OHIP-G5 [31]. In the overall evaluation of the OHIP-G5 questionnaire, the median score was three points (IQR: 5) (mean: 3.6 ± 3.6). In general, a lower score means better person-reported oral health. The OHIP-G5 total score was marginally higher in females than in males, with female participants exhibiting a slightly elevated score for orofacial pain and psychosocial influence in particular. In a review from 2009, Fillingim et al. described a finding that indicated females generally experience more pain than males. This phenomenon was also observed in the oral area [32]. A more extensive study cohort would offer further insight into disparities between the sexes in OHRQoL.

Previous studies that determined the OHIP score used the longer form, the OHIP-G14 questionnaire. The OHIP-G5 results can be compared with the results from studies in which other OHIP variants were used due to the almost identical information content [33].

Given the observation that the scale lengths of the OHIP-G5 and OHIP-G14 are not equivalent, a normalization process can be implemented to ensure the comparability of the results. To compare and interpret these, the OHIP-G5 score is multiplied by 3.5 [34] to give a mean value of 12.6 (± 12.6). In two other studies, OHIP was examined in Marfan Patients. Jenabzadeh et al. (2024) published a median OHIP-G14 score of six (IQR 15) in Marfan patients [19], while Hanisch et al. (2018) published a mean value of 13.65 (± 13.53) [14]. Hanisch et al. also showed that women (14.69 ± 14.87) had a higher OHIP-G14 total score than men (11.29 ± 9.87). This finding is consistent with the results of this study.

Accordingly, the score determined in this study lies between the previously determined scores.

A further point of interest is the comparison of the OHIP score with those of other connective tissue disorders that demonstrate some degree of phenotypical overlap of cardiovascular, skeletal, and cutaneous features [35]. In 2023, Balke et al. examined the OHIP-14 score of individuals with Ehlers-Danlos syndrome and showed a mean OHIP score of 19.6 (± 12.3) [36]. In a separate study from 2019, Nguyen et al. found a mean OHIP-14 score of 6.30 (± 6.37) for individuals with Loeys-Dietz syndrome [37]. Consequently, the OHIP score of people with Marfan syndrome in this study falls between the scores of the two other study collectives. From this it can be concluded that OHRQoL is reduced in all three connective tissue disorders even there are differences in the results between the individual differential diagnoses. In 2017, Meester et al. described the differences in manifestations of Marfan syndrome, Ehlers-Danlos syndrome and Loeys-Dietz syndrome. Among others, it has been documented that patients with Loeys-Dietz syndrome experience dental complications, including defective tooth enamel and malocclusion [35]. A specific subtype of Ehlers-Danlos syndrome has been identified as periodontal subtype, characterized by intractable, absence of attached gingiva, and pretibial plaques [35]. In hereditary diseases of the connective tissue, there are structural or regulatory defects that affect the periodontal tissue. The severity of these impairments is particularly pronounced in the periodontal subtype of Ehlers-Danlos syndrome, a condition that is associated with the development of aggressive periodontitis even in childhood. This condition is associated with a mutation in C1R and C1S, which encode proteins of the complement system and are likely involved in tissue inflammation within the periodontium [38]. Conversely, Marfan syndrome is characterized by a mutation in the fibrillin-1 protein, which is a primary component of microfibrils found in connective tissue [39]. This mutation probably leads to an altered structure of the desmodont, potentially contributing to the development of the disease. Mutations in the TGF-β signaling pathway have been identified as the underlying cause of the pathologies observed in Loeys-Dietz syndrome [40]. The potential for periodontal involvement in this disease remains speculative due to the limited available data. The precise identification of the connective tissue disorder that manifests the most pronounced symptoms in the orofacial region remains challenging because so far there are only isolated data on oral symptoms for all three syndromes, but the existing data on the OHRQoL provide a good overview and show that in connective tissue disorders much emphasis should be placed on the diagnosis and treatment in the orofacial region to increase the OHRQoL.

The Physical Oral Health Index (PhOX) can be used to determine objective oral health, with a high overall score indicating good oral health. The median PhOX total score was 79, with the minimum of the study population being 59 and the maximum 94. Compared to Reissmann et al. (2022), who found a median PhOX score of 81 (IQR: 14.5) in the general German population [23], the score of this study is only slightly lower, almost equivalent. It can therefore be concluded that there was no objective reduction in oral health in this study. The periodontium connects the teeth to the jawbone and provides structural support for the teeth within the jawbone [41]. Reissmann et al. (2022) published a mean score for condition of periodontium which was 2.5 (± 1.0) [23]. The condition of the periodontium in this study cohort is lower. During dental treatment, a particular emphasis should be placed on the regular evaluation of the periodontal health. This is due the fact that individuals suffering from periodontitis have been shown to have a higher prevalence of cardiovascular disease [42]. Therefore, it is imperative that Marfan patients not be exposed to additional risk factors because they have often cardiovascular symptoms anyway [43].

The median total PhOX score for males was 79 (IQR: 12), while that for females was 78 (IQR: 15). These values indicate that there is, in contrast to the results of the OHIP-G5, no considerable difference between the sexes. Consequently, the objectively measured oral health for male and female participants is equally good, although the females had a lower OHRQoL.

In a study in which 609 dental patients were examined, Reissmann et al. identified a correlation between OHIP and PhOX [23]. Consequently, we also hypothesized that a statistically significant correlation could be evident in our study cohort.

Since a statistically significant correlation could not be identified between the OHIP-G5 and the PhOX scores, it indicates that despite the participants´ perception of reduced OHRQoL, their objective measured oral health (PhOX) remain unaltered. A more substantial sample size would offer additional insights into possible correlations. Given the multitude of published anatomical alterations in the maxillofacial area (for example dolichocephaly, retrognathia, long face, and a high narrow palatal arch) [15], it is plausible that individuals with Marfan syndrome may perceive their oral health to be inferior, consequently leading to a diminished OHRQoL that does not correlate significantly with objectively measured oral health. Psychosocial factors such as experiences of stigmatization, a high disease burden and insecurities in social interaction could have a detrimental effect on OHRQoL. The medical context in which some of those affected live, which is characterized by frequent visits to the doctor, surgical interventions and a heightened awareness of their own physical condition, could cause an increased sensitivity to perceived abnormalities. This phenomenon could be exacerbated by the lack of interdisciplinary collaboration between medical disciplines, which could lead to a sense of isolation and increased psychological distress in people dealing with dental problems.

To date, there are no specific treatment recommendations for the dental treatment of Marfan patients. They should be treated individually according to their symptoms. Interdisciplinary treatment may be necessary. The orthodontist and dentist should work closely together to ensure optimal treatment.

It is imperative that dental professionals prioritize oral hygiene in patients with Marfan syndrome, routinely assess periodontal health, and advise patients on appropriate oral hygiene measures. It is imperative to emphasize the importance of periodontal disease prevention practices, such as regular interdental cleaning at home. Based on the results of this study, a future direction for research in this area could entail the formulation of comprehensive treatment guidelines for patients diagnosed with Marfan syndrome.

A data collection with a larger study cohort would be desirable in order to make a precise treatment recommendation and further investigate the reduced OHRQoL. This would allow for the identification of additional correlations.

### Limitations

The study and the results are limited, among other things, by the small study cohort. Due to the rarity of the disease, it was only possible to recruit 29 people for the study. A larger number of participants would have allowed more precise statements about a possible statistically significant correlation. Furthermore, one bias of the study is that most of the participants are involved in the self-help group Marfan Hilfe e.V., which means that it can be assumed that the participants are interested in the optimal treatment of their disease and tend to have more regular routine check-ups and treatments at the dental professional, as it is recommended by the self-help group and experts. Accordingly, it would also be advantageous to examine people outside the self-help group. Potential cofounding factors, such as participants´ socioeconomic status and oral hygiene habits, were not taken into account in the data collection and should be examined more closely in future studies. Taking X-rays would have provided additional information about possible pathologies. However, since radiation protection was a priority and the patients had not presented with acute dental complaints but specifically for the purpose of data collection for the study, there was no indication for taking X-rays. A potential bias of the study results from the linguistic limitation to German-speaking participants. As a result, Marfan patients who do not speak German could not be included. This limits the generalizability to multilingual or international populations.

## Conclusions

This study showed that Marfan patients have a poorer oral quality of life than the general population. However, the objectively measured oral health was only slightly worse than that of the comparison data. Particular attention should be paid to the condition of the periodontium. No statistically significant correlation was found between the person-reported measured OHRQoL and the objectively measured oral health (PhOX). The hypothesis that optimal oral health with an enhanced oral health related quality of life (OHRQoL) does not appear to be applicable in the context of Marfan syndrome. In summary, it is nevertheless recommended that Marfan patients pay close attention to oral hygiene and have their oral health checked regularly by dental professionals. Interdisciplinary cooperation between dentists and orthodontists should take place during check-ups in order to provide adequate dental care for the anatomical and clinical pathologies in Marfan patients. A larger study group would provide further information on the effects of Marfan syndrome.

## Data Availability

The datasets used and/or analyzed during the current study are available from the corresponding author on reasonable request.

## Abbreviations

IQR: Interquartile range
SD: Standard deviation
OHIP: Oral Health Impact Profile
OHRQoL: Oral Health-Related Quality of Life
PhOX: Physical Oral Health Index
